# Supplementary material for: Meta-analyses identify DNA methylation associated with kidney function and damage
Source: Nat Commun. 2021 Dec 9;12:7174. doi: 10.1038/s41467-021-27234-3 (PMC8660832; doi:10.1038/s41467-021-27234-3)
Supplement: Supplementary file 1 — Supplementary Information [file 41467_2021_27234_MOESM1_ESM.pdf]

Meta-analyses identify DNA methylation associated with kidney function and damage

Schlosser *et al.*

SUPPLEMENTARY INFORMATION

Supplementary Note: Extended acknowledgements 3

Supplementary Figures 13

Supplementary Figure 1: Overview of the project..13
Supplementary Figure 2: Forest plot of UACR association cg22304262 14
Supplementary Figure 3: EWAS results of CKD and microalbuminuria 15
Supplementary Figure 4: Lookup of the EWAS results in a cohort of CKD patients 16
Supplementary Figure 5: Regional association plots of two showcase CpGs..17
Supplementary Figure 6: DNA methylation effects in kidney tissue. 20
Supplementary Figure 7: Mendelian randomization plots 21
Supplementary Figure 8: BACON plots 23
Supplementary Figure 9: Schoenfeld residuals of the time-to-event analyses in CKD patients 24

Supplementary Note: Extended acknowledgements

CpG probe annotation was supported by software resources provided via the Caché Campus program of the InterSystems Corporation (Cambridge, MA, USA) to Alexander Teumer.

Extended acknowledgements and funding sources

ARIC

The Atherosclerosis Risk in Communities study has been funded in whole or in part with Federal funds from the National Heart, Lung, and Blood Institute, National Institutes of Health, Department of Health and Human Services (contract numbers HHSN268201700001I, HHSN268201700002I, HHSN268201700003I, HHSN268201700004I and HHSN268201700005I), R01HL087641, R01HL059367 and R01HL086694; National Human Genome Research Institute contract U01HG004402; and National Institutes of Health contract HHSN268200625226C. Funding was also supported by 5RC2HL102419, R01NS087541 and R01HL131136. The authors thank the staff and participants of the ARIC study for their important contributions. Infrastructure was partly supported by Grant Number UL1RR025005, a component of the National Institutes of Health and NIH Roadmap for Medical Research. The work of Anna Köttgen was funded by the Deutsche Forschungsgemeinschaft (DFG, German Research Foundation) 3598/5-1 (Heisenberg professorship), Project-ID 192904750 – CRC 992 Medical Epigenetics and Project-ID 431984000 – CRC 1453 Nephrogenetics. Adrienne Tin is supported by NIAMS grant R01AR073178-01A1. The work of Pascal Schlosser was funded by DFG Project-ID 192904750 – CRC 992 Medical Epigenetics, by DFG grant SCHL 2292/1-1 (Walter Benjamin Fellowship), and the EQUIP Program for Medical Scientists, Faculty of Medicine, University of Freiburg.

CARDIA

The Coronary Artery Risk Development in Young Adults Study (CARDIA) is conducted and supported by the National Heart, Lung, and Blood Institute (NHLBI) in collaboration with the University of Alabama at Birmingham (HHSN268201800005I &

HHSN268201800007I), Northwestern University (HHSN268201800003I), University of Minnesota (HHSN268201800006I), and Kaiser Foundation Research Institute (HHSN268201800004I). This manuscript has been reviewed by CARDIA for scientific content.

CHS

This CHS research was supported by NHLBI contracts HHSN268201200036C, HHSN268200800007C, HHSN268201800001C, N01HC55222, N01HC85079, N01HC85080, N01HC85081, N01HC85082, N01HC85083, N01HC85086, 75N92021D00006; and NHLBI grants U01HL080295, R01HL087652, R01HL105756, R01HL103612, R01HL120393, R01HL085251, and U01HL130114 with additional contribution from the National Institute of Neurological Disorders and Stroke (NINDS). Additional support was provided through R01AG023629 from the National Institute on Aging (NIA). A full list of principal CHS investigators and institutions can be found at CHS-NHLBI.org.

The provision of genotyping data was supported in part by the National Center for Advancing Translational Sciences, CTSI grant UL1TR001881, and the National Institute of Diabetes and Digestive and Kidney Disease Diabetes Research Center (DRC) grant DK063491 to the Southern California Diabetes Endocrinology Research Center.

The content is solely the responsibility of the authors and does not necessarily represent the official views of the National Institutes of Health.

EstBB

The Estonian Biobank is a population-based biobank of the Estonian Genome Center at the University of Tartu (EstBB). The samples used in this study were selected from the EstBB Center for Translational Genomics (CTG) cohort of individuals who have been recontacted for a second time-point sample (EstBB-CTG), and an aging study of 50 younger (age 22–34) and 50 older (age 73–84) individuals (EstBB-YO). The EstBB research was supported by the European Union through the European Regional Development Fund (Project No. 2014-2020.4.01.15-

	0012). Data analyzes were carried out in part in the High-Performance Computing Center of University of Tartu.
ESTHER	The ESTHER study was supported by the Baden-Württemberg State Ministry of Science, Research and Arts (Stuttgart, Germany), the Federal Ministry of Education and Research (Berlin, Germany) and the Federal Ministry of Family Affairs, Senior Citizens, Women and Youth (Berlin, Germany). ESTHER is a statewide population-based cohort study conducted in Saarland/Germany. 9,940 older adults (50-75 years) were recruited in 2000-2002 and have been regularly followed up since then. Recruitment and collection of baseline data used for this study have been supported by a grant from the Baden-Württemberg state Ministry of Science, Research and Arts. This study included a random sample of ESTHER participants (ESTHERa, ESTHERb) for whom epigenome wide DNA profiling was performed.
FHS	FHS is funded by the U.S. NIH contract N01-HC-25195 and HHSN268201500001I.
GCKD	The GCKD study is supported by the German Ministry of Education and Research (Bundesministerium für Bildung und Forschung, FKZ 01ER 0804, 01ER 0818, 01ER 0819, 01ER 0820, and 01ER 0821), KfH Foundation for Preventive Medicine, Innovative Medicines Initiative 2 Joint Undertaking (BEAt-DKD, grant number 115974) and corporate sponsors (www.gckd.org).
GENOA	Support for the Genetic Epidemiology Network of Arteriopathy (GENOA) was provided by the National Heart, Lung and Blood Institute (U01 HL054457, RC1 HL100185, R01 HL119443, and R01 HL133221) and the National Institute of Diabetes and Digestive and Kidney Diseases (R01 DK073537) of the National Institutes of Health. We appreciate technical assistance from Stephen T. Turner, Pamela I. Hammond, Julie M. Cunningham, and the Mayo Clinic Advanced Genomics Technology Center. We would also like to thank the families that participated in the GENOA study.

GS:SFHS This work was supported by a Wellcome Trust Strategic Award "STratifying Resilience and Depression Longitudinally" (STRADL) [104036/Z/14/Z]. Generation Scotland received core support from the Chief Scientist Office of the Scottish Government Health Directorates [CZD/16/6] and the Scottish Funding Council [HR03006] and is currently supported by the Wellcome Trust [216767/Z/19/Z]. We are grateful to all the families who took part, the general practitioners and the Scottish School of Primary Care for their help in recruiting them, and the whole Generation Scotland team. Ethics approval for the study was given by the NHS Tayside committee on research ethics (reference 05/S1401/8).

JHS The Jackson Heart Study (JHS) is supported and conducted in collaboration with Jackson State University (HHSN268201800013I), Tougaloo College (HHSN268201800014I), the Mississippi State Department of Health (HHSN268201800015I) and the University of Mississippi Medical Center (HHSN268201800010I, HHSN268201800011I and HHSN268201800012I) contracts from the National Heart, Lung, and Blood Institute (NHLBI) and the National Institute on Minority Health and Health Disparities (NIMHD). The authors also wish to thank the staffs and participants of the JHS.

KORA F4 The KORA research platform (KORA, Cooperative Health Research in the Region of Augsburg) was initiated and financed by the Helmholtz Zentrum München - German Research Center for Environmental Health, which is funded by the German Federal Ministry of Education and Research and by the State of Bavaria. Furthermore, KORA research was supported within the Munich Center of Health Sciences (MC Health), Ludwig-Maximilians-Universität, as part of LMUinnovativ. Methylation analysis was performed at the Core Facility Genotyping (Genome Analysis Center (GAC), Helmholtz Zentrum München) under the supervision of Dr. Jennifer Kriebel and Dr. Eva Reischl. The German Diabetes Center (DDZ) is supported by the Ministry of Culture and Science of the State of North Rhine-Westphalia and the German Federal Ministry of Health. This

	study was supported in part by a grant from the German Federal Ministry of Education and Research to the German Center for Diabetes Research (DZD).
Lothian Birth Cohorts of 1921 and 1936	The LBC1921 was supported by the UK's Biotechnology and Biological Sciences Research Council (BBSRC), a Royal Society–Wolfson Research Merit Award to I.J.D., and the Chief Scientist Office (CSO) of the Scottish Government's Health Directorates. The LBC1936 is supported by Age UK (Disconnected Mind project, which supports S.E.H.), the Medical Research Council (G0701120, G1001245, MR/M013111/1, MR/R024065/1, which supports S.R.C.), and the University of Edinburgh. Methylation typing in both the LBC1921 and LBC1936 was supported by Centre for Cognitive Ageing and Cognitive Epidemiology (Pilot Fund award), Age UK, The Wellcome Trust Institutional Strategic Support Fund, The University of Edinburgh, and The University of Queensland.
LifeLinesDeep	The work was supported by the Dutch Digestive Disease Foundation (MLDS WO11-30 to CW), the European Research Council Advanced Grant (ERC-671274 to CW), the Netherlands Organization for Scientific Research (NWO-VENI grant 916-10135 to LF and NWO VIDI grant 917-14374 to LF) and the European Union's Seventh Framework Programme (EU FP7) TANDEM project (HEALTH-F3-2012-305279 to CW).
LOLIPOP	The LOLIPOP study is supported by the National Institute for Health Research (NIHR) Comprehensive Biomedical Research Centre Imperial College Healthcare NHS Trust, the British Heart Foundation (SP/04/002), the Medical Research Council (G0601966, G0700931), the Wellcome Trust (084723/Z/08/Z, 090532 & 098381) the NIHR (RP-PG-0407-10371), the NIHR Official Development Assistance (ODA, award 16/136/68), the European Union FP7 (EpiMigrant, 279143) and H2020 programs (iHealth-T2D, 643774). We acknowledge support of the MRC-PHE Centre for Environment and Health, and the NIHR Health Protection Research Unit on Health Impact of Environmental Hazards. The work was carried out in part at the

NIHR/Wellcome Trust Imperial Clinical Research Facility. The views expressed are those of the author(s) and not necessarily those of the Imperial College Healthcare NHS Trust, the NHS, the NIHR or the Department of Health. We thank the participants and research staff who made the study possible. JC is supported by the Singapore Ministry of Health's National Medical Research Council under its Singapore Translational Research Investigator (STaR) Award (NMRC/STaR/0028/2017).

LURIC	LURIC was supported by the 7th Framework Program of the EU (AtheroRemo, grant agreement number 201668 and RiskyCAD, grant agreement number 305739). The work of W.M., M.E.K. and S.L. is supported as part of the Competence Cluster of Nutrition and Cardiovascular Health (nutriCARD) Halle-Jena-Leipzig (Germany) which is funded by the German Ministry of Education and Research (grant agreement numbers 01EA1808A and 01EA1411A). The authors thank the LURIC study team who were involved in patient recruitment as well as sample and data handling, in addition to the laboratory staff at the Ludwigshafen General Hospital and the universities of Freiburg, Ulm and Heidelberg, Germany.
MESA	MESA and the MESA SHARe project are conducted and supported by the National Heart, Lung, and Blood Institute (NHLBI) in collaboration with MESA investigators. Support for MESA is provided by contracts 75N92020D00001, HHSN268201500003I, N01-HC-95159, 75N92020D00005, N01-HC-95160, 75N92020D00002, N01-HC-95161, 75N92020D00003, N01-HC-95162, 75N92020D00006, N01-HC-95163, 75N92020D00004, N01-HC-95164, 75N92020D00007, N01-HC-95165, N01-HC-95166, N01-HC-95167, N01-HC-95168, N01-HC-95169, UL1-TR-000040, UL1-TR-001079, UL1-TR-001420, UL1-TR-001881, and DK063491. The MESA Epigenomics & Transcriptomics Studies were funded by NIH grants R01HL101250, R01HL119962, R01DK101921, R01HL135009, and 1RF1AG054474.

Normative Aging Study	The molecular analyses in the US Department of Veterans Affairs (VA) Normative Aging Study have been supported by the U.S. National Institute of Environmental Health Sciences (NIEHS) (R01ES015172, R01ES021733). The VA Normative Aging Study is supported by the Cooperative Studies Program/ERIC, US Department of Veterans Affairs, and is a research component of the Massachusetts Veterans Epidemiology Research and Information Center (MAVERIC). Additional support to the VA Normative Aging Study was provided by the US Department of Agriculture, Agricultural Research Service (contract 53-K06-510). The views expressed in this paper are those of the authors and do not necessarily represent the views of the US Department of Veterans Affairs.
NFBC1966	MW was supported by the European Union's Horizon 2020 research and innovation programme under grant agreement No 633212. NFBC1966 received financial support from the Academy of Finland (project grants 104781, 120315, 129269, 1114194, 24300796, Center of Excellence in Complex Disease Genetics and SALVE), University Hospital Oulu, Biocenter, University of Oulu, Finland (75617), NHLBI grant 5R01HL087679-02 through the STAMPEED program (1RL1MH083268-01), NIH/NIMH (5R01MH63706:02), ENGAGE project and grant agreement HEALTH-F4-2007-201413, EU FP7 EurHEALTHAgeing -277849, the Medical Research Council, UK (G0500539, G0600705, G1002319, PrevMetSyn/SALVE) and the MRC, Centenary Early Career Award. The program is currently being funded by the H2020-633595 DynaHEALTH action, academy of Finland EGEA-project (285547) and EU H2020 ALEC project (Grant Agreement 633212).
PIVUS	The PIVUS study was funded by Uppsala University Hospital.
Rhineland Study	The Rhineland Study is supported by the German Center for Neurodegenerative Diseases (DZNE). The omics analyses in the Rhineland Study were partly supported by the Diet-Body-Brain Competence Cluster in Nutrition Research funded by the

	Federal Ministry of Education and Research (grant number 01EA1410C and FKZ: 01EA1809C).
RODAM	The RODAM study was supported by the European Commission under the Framework Programme (Grant Number: 278901). A.A.A. and K.A.C.M. are supported by the Intramural Research Program of the National Institutes of Health in the Center for Research on Genomics and Global Health (CRGGH). The CRGGH is supported by the National Human Genome Research Institute, the National Institute of Diabetes and Digestive and Kidney Diseases, the Center for Information Technology, and the Office of the Director at the National Institutes of Health (1ZIAHG200362). The authors are grateful to the RODAM advisory board members for their valuable support in shaping the methods, to the research assistants, interviewers and other staff of the five research locations who have taken part in gathering the data and, most of all, to the Ghanaian volunteers participating in this project. We gratefully acknowledge J. van Straalen from the Department of Clinical Chemistry, Amsterdam University Medical Centers (Amsterdam, the Netherlands) for his valuable support with standardisation of the laboratory procedures, and the Academic Medical Center (AMC) Biobank for support in biobank management and storage of collected samples.
Rotterdam Study	The Rotterdam Study is funded by Erasmus Medical Center and Erasmus University, Rotterdam, Netherlands Organization for the Health Research and Development (ZonMw), the Research Institute for Diseases in the Elderly (RIDE), the Ministry of Education, Culture and Science, the Ministry for Health, Welfare and Sports, the European Commission (DG XII), and the Municipality of Rotterdam. The authors are grateful to the Rotterdam Study participants, the staff involved with the Rotterdam Study and the participating general practitioners and pharmacists.<br><br>The generation and management of the Illumina 450K methylation array data (EWAS data) for the Rotterdam Study

was executed by the Human Genotyping Facility of the Genetic Laboratory of the Department of Internal Medicine, Erasmus MC, the Netherlands. The EWAS data was funded by the Genetic Laboratory of the Department of Internal Medicine, Erasmus MC, and by the Netherlands Organization for Scientific Research (NWO; project number 184021007) and made available as a Rainbow Project (RP3; BIOS) of the Biobanking and Biomolecular Research Infrastructure Netherlands (BBMRI-NL). We thank Mr. Michael Verbiest, Ms. Mila Jhamai, Ms. Sarah Higgins, Mr. Marijn Verkerk, and Lisette Stolk for their help in creating the methylation database. We thank Pascal Arp, Mila Jhamai, MarijnVerkerk, Lizbeth Herrera and Marjolein Peters for their help in creating the GWAS database.

SHIP-Trend	SHIP is part of the Community Medicine Research net of the University of Greifswald, Germany, which is funded by the Federal Ministry of Education and Research (grants no. 01ZZ9603, 01ZZ0103, and 01ZZ0403), the Ministry of Cultural Affairs as well as the Social Ministry of the Federal State of Mecklenburg-West Pomerania, and the network 'Greifswald Approach to Individualized Medicine (GANI_MED)' funded by the Federal Ministry of Education and Research (grant 03IS2061A). DNA methylation data have been supported by the DZHK (grant 81X3400104). The SHIP authors are grateful to Paul S. DeVries for his support with the EWAS pipeline.
SKIPOGH	The SKIPOGH study is supported by a grant from the Swiss national science foundation (FN33CM30-124087).
Strong Heart Study	The Strong Heart Study was supported by grants from the National Heart, Lung, and Blood Institute (NHLBI) (contract numbers 75N92019D00027, 75N92019D00028, 75N92019D00029 and 75N92019D00030) and previous grants (R01HL090863, R01HL109315, R01HL109301, R01HL109284, R01HL109282, and R01HL109319 and cooperative agreements: U01HL41642, U01HL41652, U01HL41654, U01HL65520 and U01HL65521) and by the National Institute of Environmental Health Sciences (grant numbers R01ES021367,

R01ES025216, R01ES032638, P42ES010349, P30ES009089). Arce Domingo also received the support of a fellowship from "la Caixa" Foundation (ID 100010434) (fellowship code "LCF/BQ/DR19/11740016").

YFS	The Young Finns Study has been financially supported by the Academy of Finland: grants 322098, 286284, 134309 (Eye), 126925, 121584, 124282, 129378 (Salve), 117787 (Gendi), and 41071 (Skidi); the Social Insurance Institution of Finland; Competitive State Research Financing of the Expert Responsibility area of Kuopio, Tampere and Turku University Hospitals (grant X51001); Juho Vainio Foundation; Paavo Nurmi Foundation; Finnish Foundation for Cardiovascular Research; Finnish Cultural Foundation; The Sigrid Juselius Foundation; Tampere Tuberculosis Foundation; Emil Aaltonen Foundation; Yrjö Jahnsson Foundation; Signe and Ane Gyllenberg Foundation; Diabetes Research Foundation of Finnish Diabetes Association; EU Horizon 2020 (grant 755320 for TAXINOMISIS); This project has received funding from the European Union's Horizon 2020 research and innovation programme under grant agreement No 848146; European Research Council (grant 742927 for MULTIEPIGEN project); Tampere University Hospital Supporting Foundation and Finnish Society of Clinical Chemistry.

Josine L. Min is supported by the UK Medical Research Council Integrative Epidemiology Unit at the University of Bristol (MC_UU_00011/5).
Gibran Hemani is funded by the Wellcome Trust and the Royal Society (208806/Z/17/Z).

Estonian Biobank Research Team: group author acknowledgement
Tõnu Esko[1], Andres Metspalu[1], Reedik Mägi[1], Mari Nelis[1]
[1]Estonian Biobank, Institute of Genomics, University of Tartu, Tartu, Estonia

Supplementary Figures

Supplementary Figure 1: Overview of the project

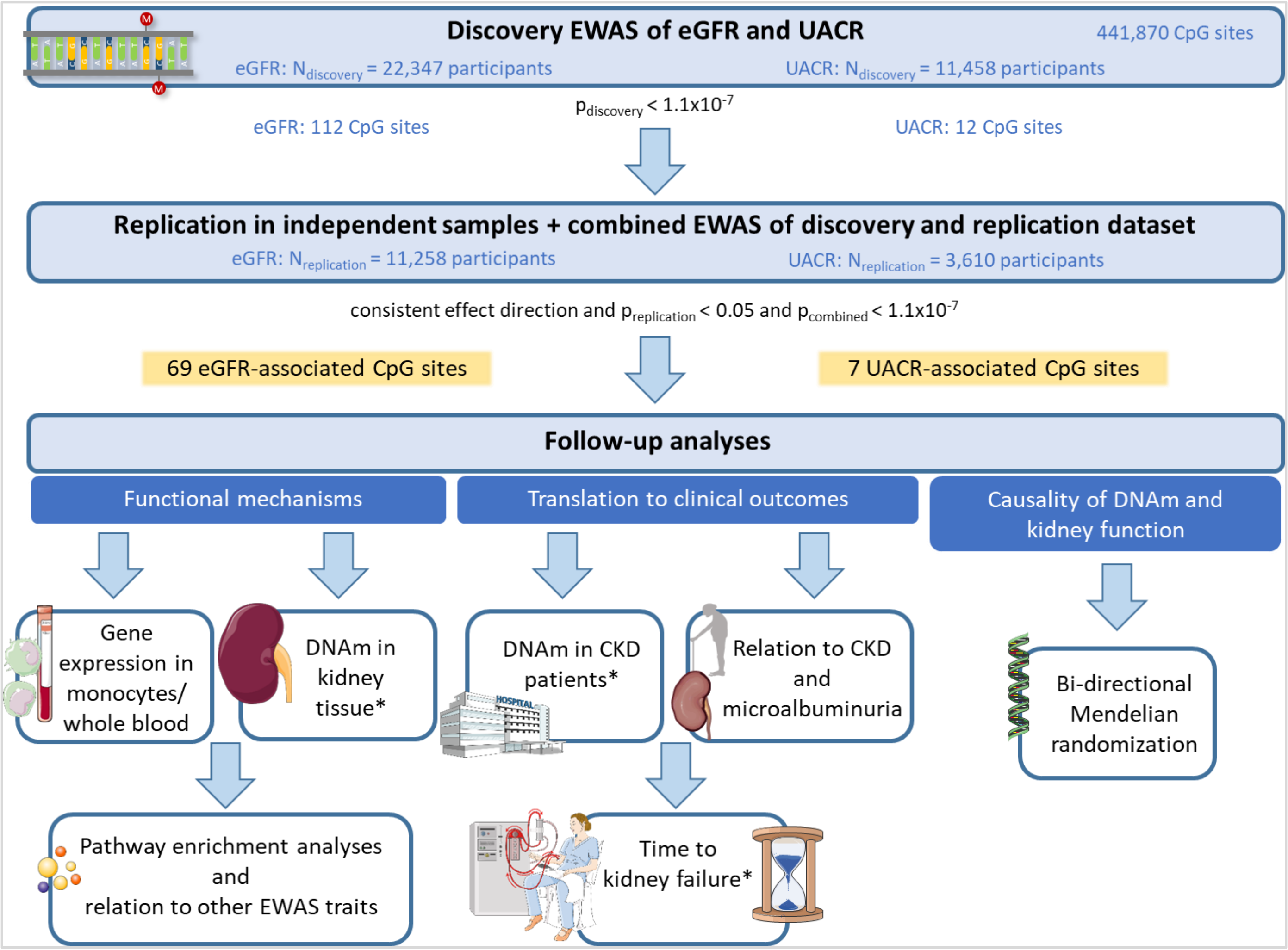

Icon credit: Servier Medical Art by Servier (licensed under a Creative Commons Attribution 3.0 Unported License).

Overview of the analyses conducted within the project.

* Analysis performed for eGFR-associated CpG sites only

EWAS: Epigenome-wide Association Study; CKD: chronic kidney disease; eGFR: estimated glomerular filtration rate; UACR: urinary albumin-to-creatinine ratio

Supplementary Figure 2: Forest plot of UACR association cg22304262

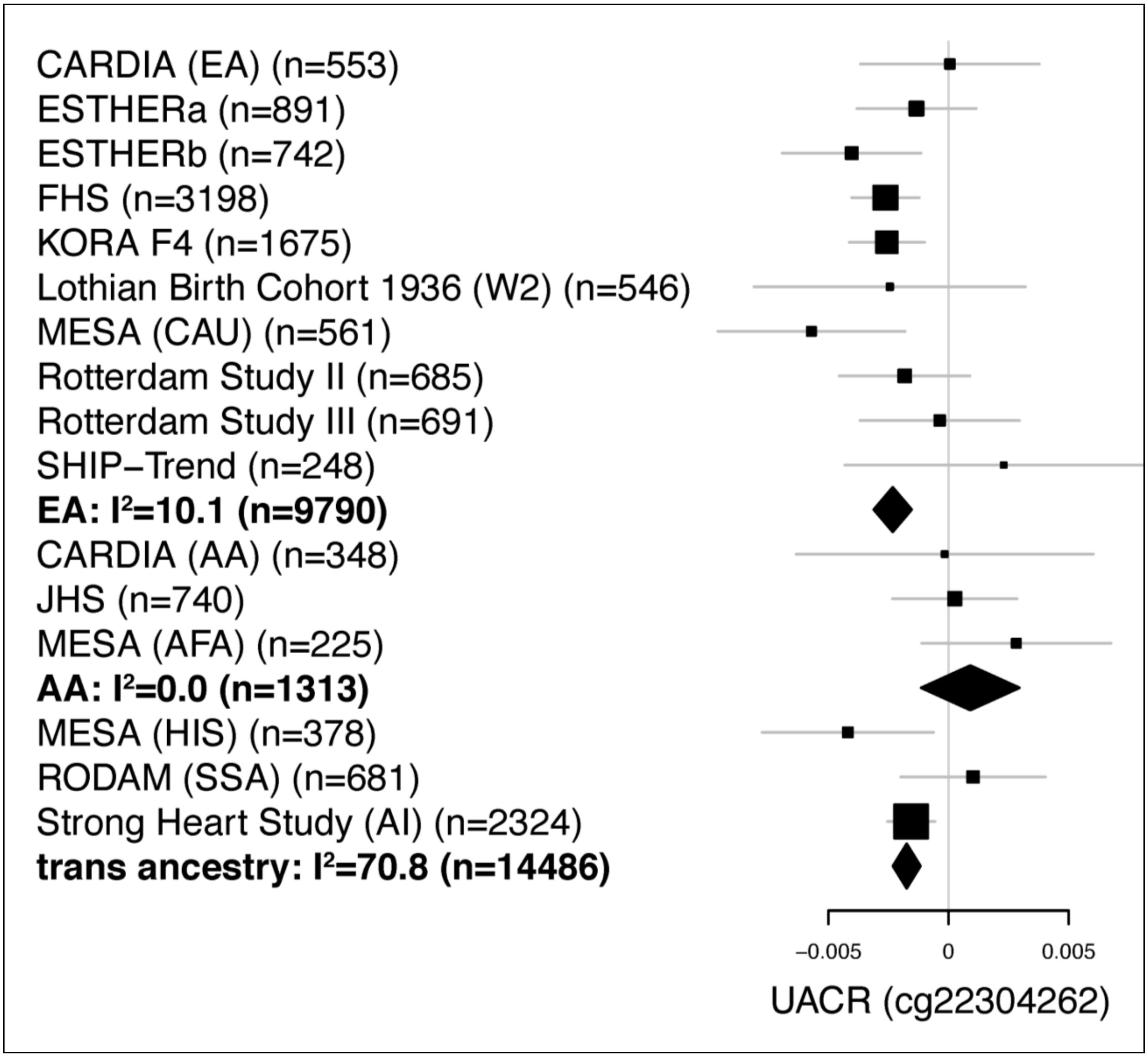

Trans-ethnic forest plot of the UACR-associated CpG site cg22304262. The error bars indicate the 95% confidence intervals.

AA: African American ancestry; EA: European ancestry; HIS: Hispanics; SSA: Sub-Saharan African ancestry; American Indian ancestry; UACR: urinary albumin-to-creatinine ratio

Supplementary Figure 3: EWAS results of CKD and microalbuminuria

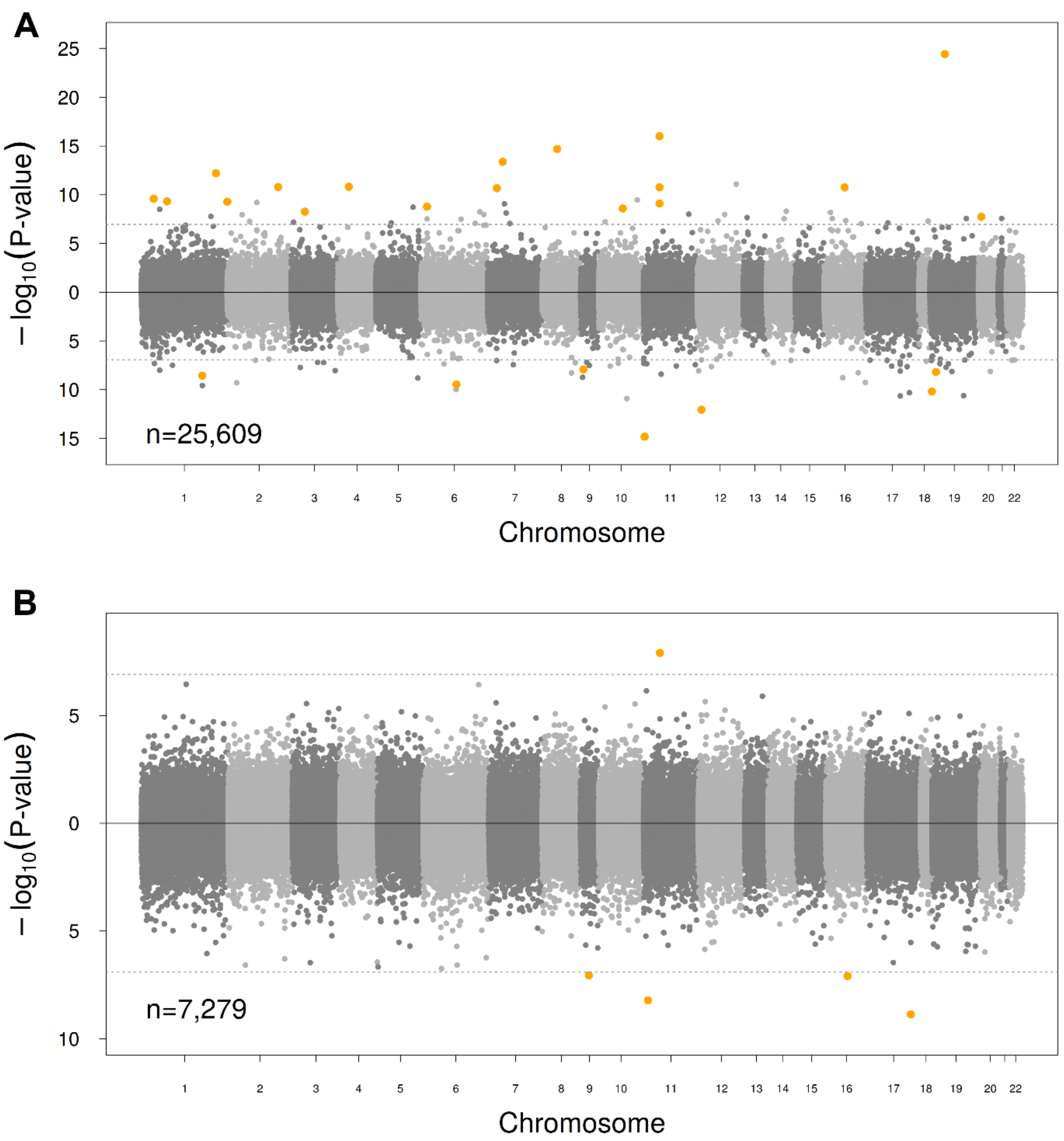

Chicago plots of the Epigenome-wide Association Study (EWAS) results for chronic kidney disease (CKD) (A) and microalbuminuria (B) using the combined discovery and replication sample. The sites are ordered by their chromosomal position on the x-axis, with their $-\log_{10}$ p-value of the association Wald-test provided on the y-axis. CpG sites having positive correlation with the trait are plotted in the upper part, sites with negative correlation in the lower part. The dotted horizontal lines

represent the level of significance (p<1.1E-7). Replicated sites are colored in orange.

Supplementary Figure 4: Lookup of the EWAS results in a cohort of CKD patients

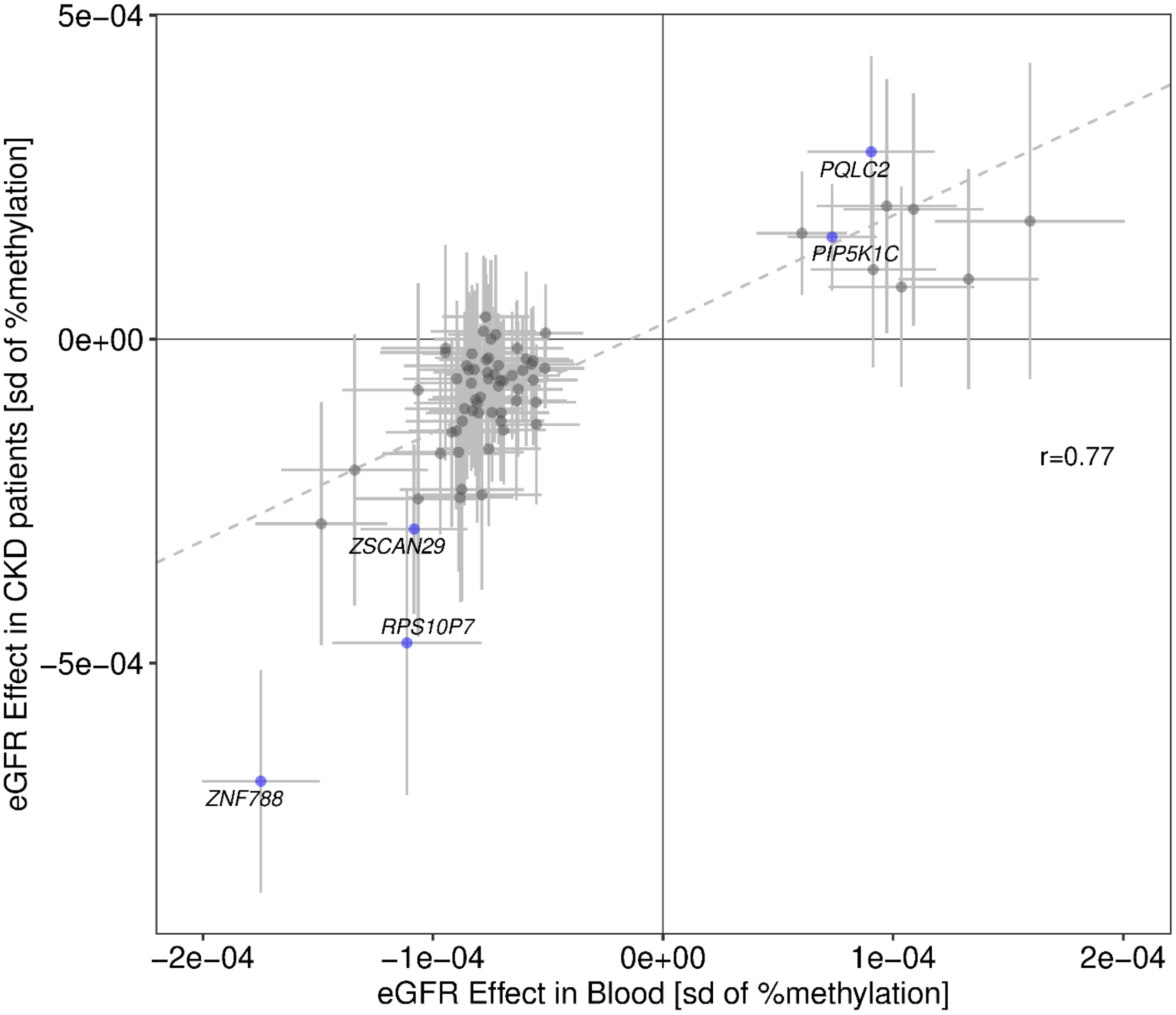

Comparison of effect estimates of the association Wald-test of the significant estimated glomerular filtration rate (GFR)-associated CpG sites obtained from population-based cohorts (n=33,605) (x-axis) with their corresponding effect size among chronic kidney disease (CKD) patients (n=559) participating in the GCKD cohort (y-axis). Sites that were significantly associated (Bonferroni corrected p-value <0.05/69) are colored in blue and labeled with the closest gene name. The dashed line represents the linear regression slope between the dots. Error bars indicate the 95% confidence intervals, and the Pearson correlation coefficient r between the effect estimates is shown.

Supplementary Figure 5: Regional association plots of two showcase CpGs

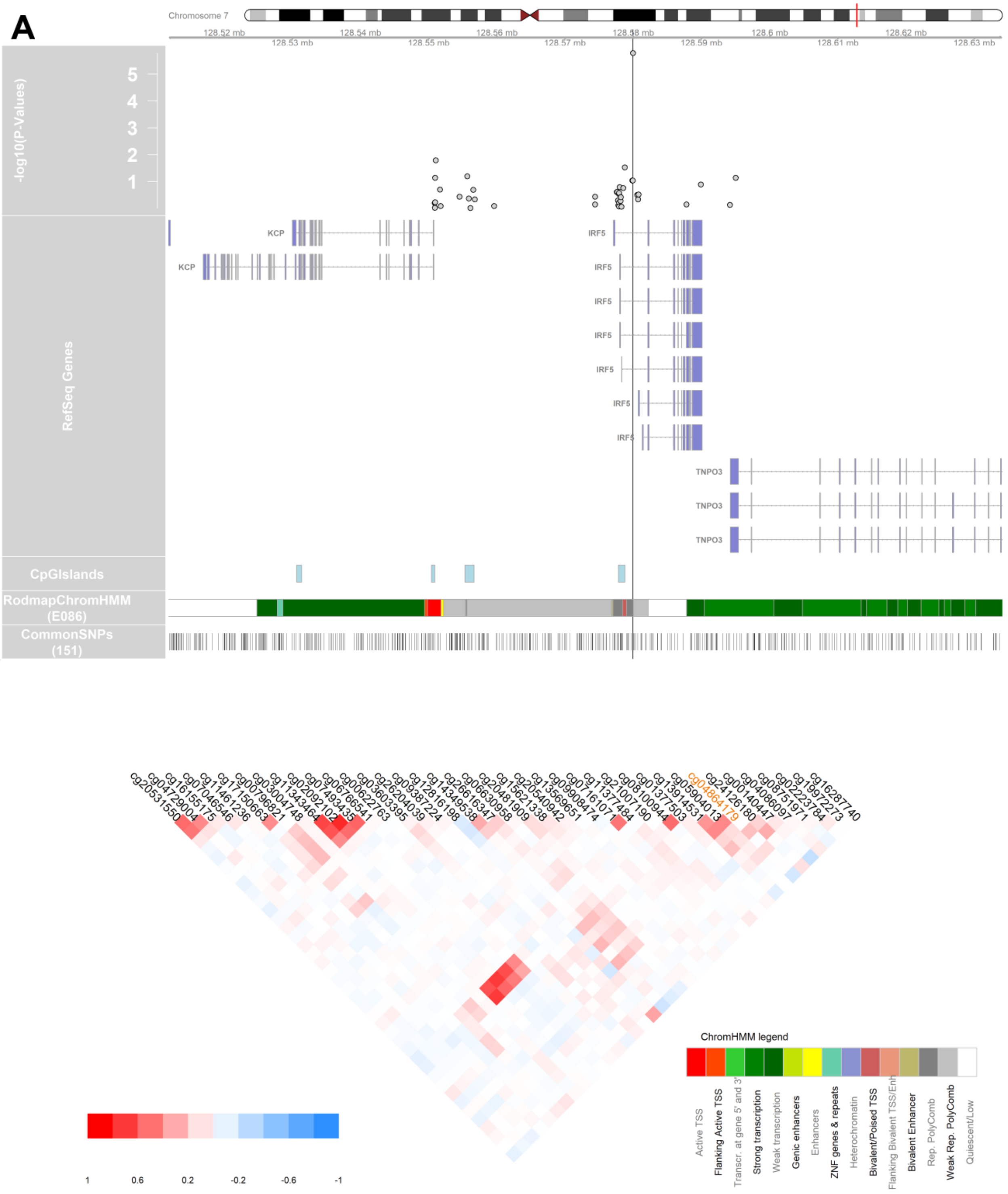

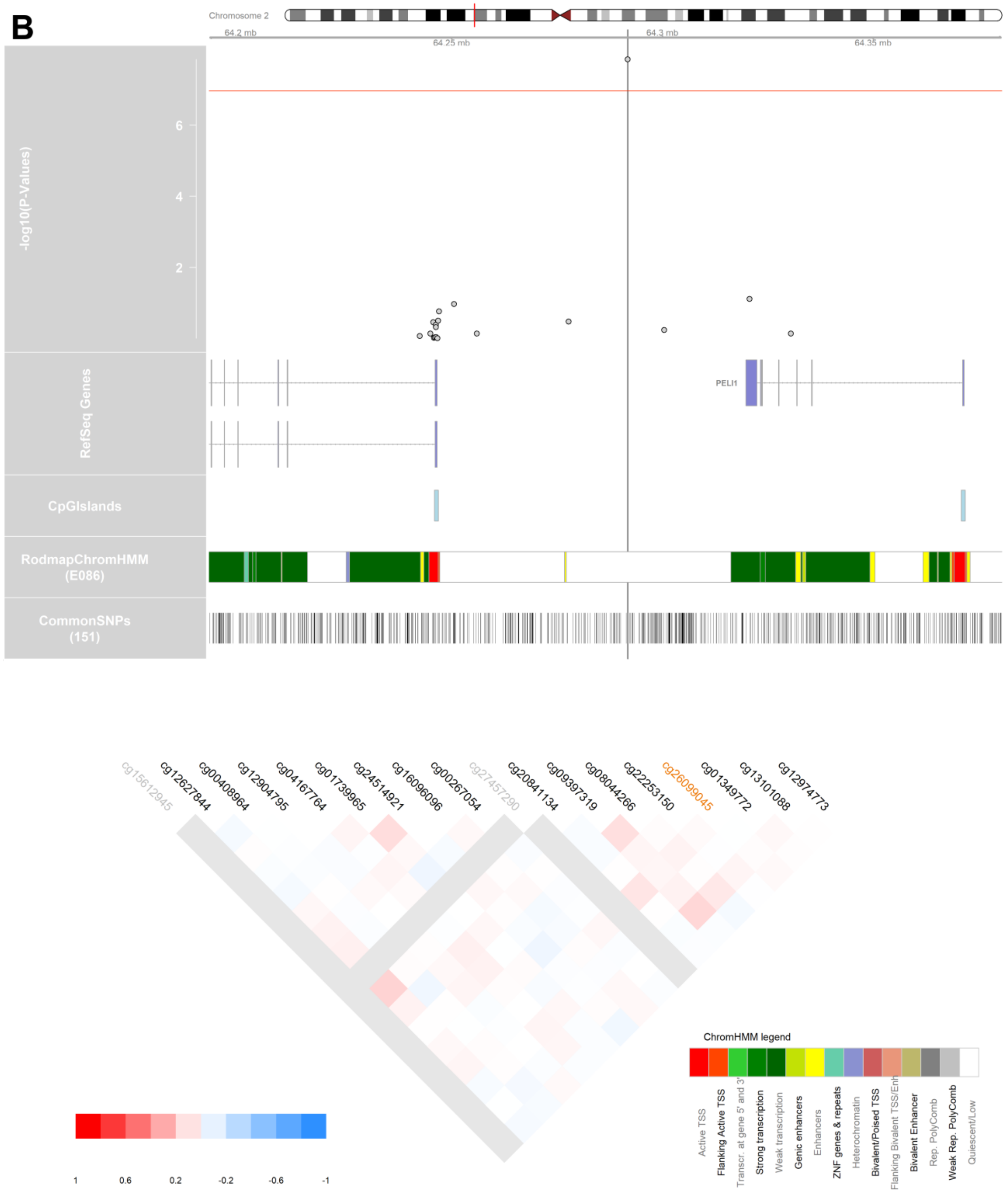

Regional association plots are provided for two of the estimated glomerular filtration rate (GFR)-associated results: cg04864179 in *IRF5* (A) that was associated with mRNA in blood and significant in the Mendelian randomization analysis on eGFR, and cg26099045 near *PELI1* (B) that was associated with eGFR and fibrosis

in kidney tissue. In both panels, the upper part shows the association results in the region of the CpG including the genes in vicinity and DNA-related annotations. The ChromHMM annotation information is provided for the fetal kidney epigenome. The lower part displays a correlation map of the nearby CpG based on the data of the KORA study DNA methylation samples.

Supplementary Figure 6: DNA methylation effects in kidney tissue.

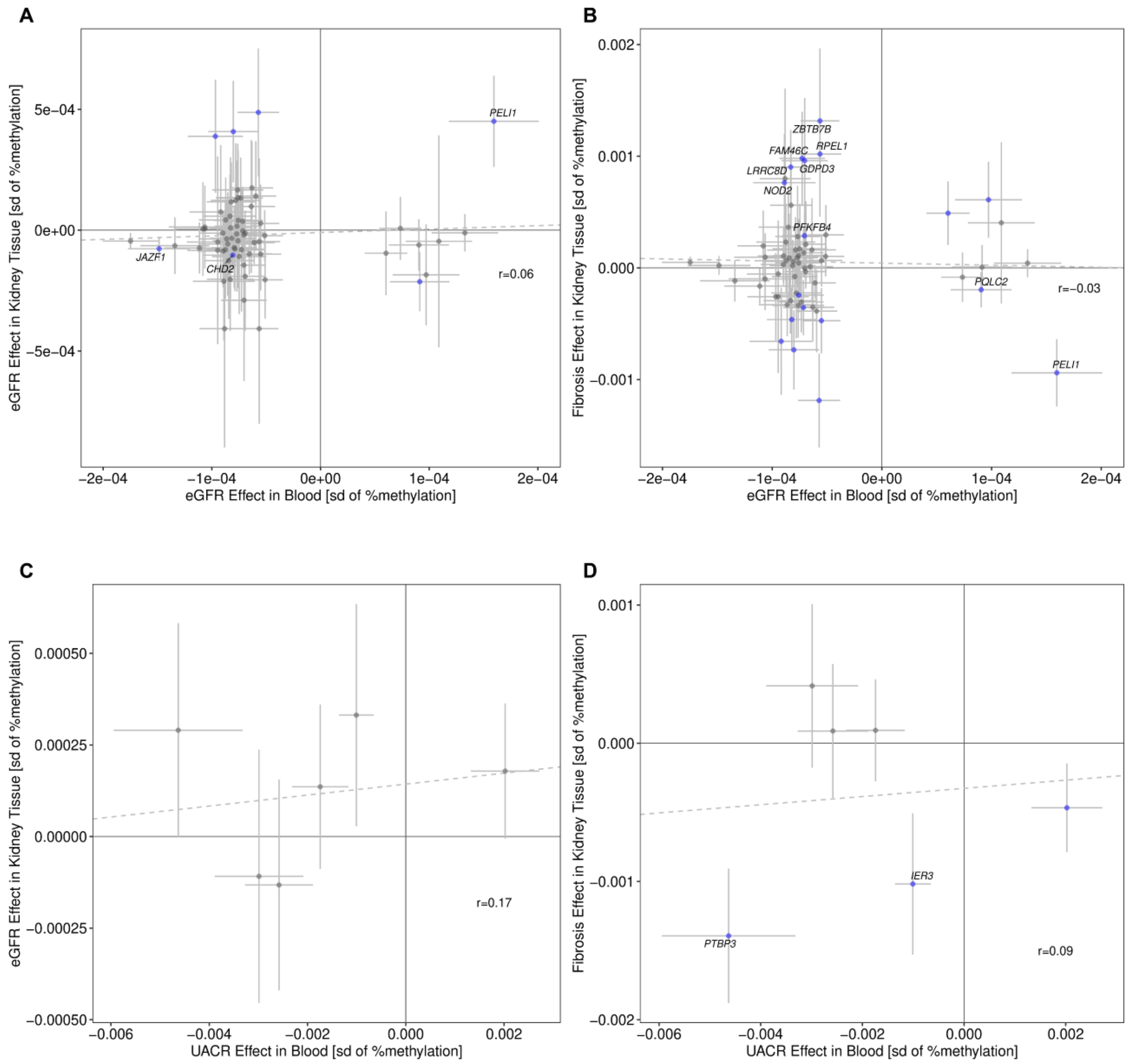

Comparison of effect estimates of the association Wald-test of the significantly associated CpG sites for estimated glomerular filtration rate (GFR) (n=33,605) (A-B) and urinary albumin-to-creatinine ratio (UACR) (n=15,068) (C-D) with the effect on eGFR (A,C) and fibrosis (B,D) when quantified from kidney tissue (n=506). The effects in the combined epigenome-wide association study (x-axis) are compared with the corresponding DNA methylation effects in the kidney tissue samples (y-axis). Sites that were significantly associated (false discovery rate < 0.05) in kidney

tissue are colored in blue, and labeled with the closest gene name if the effect direction was consistent with the blood samples. The dashed line represents the linear regression slope between the dots. In all panels, error bars indicate the 95% confidence intervals, and the Pearson correlation coefficient r between the effect estimates is shown.

Supplementary Figure 7: Mendelian randomization plots

A

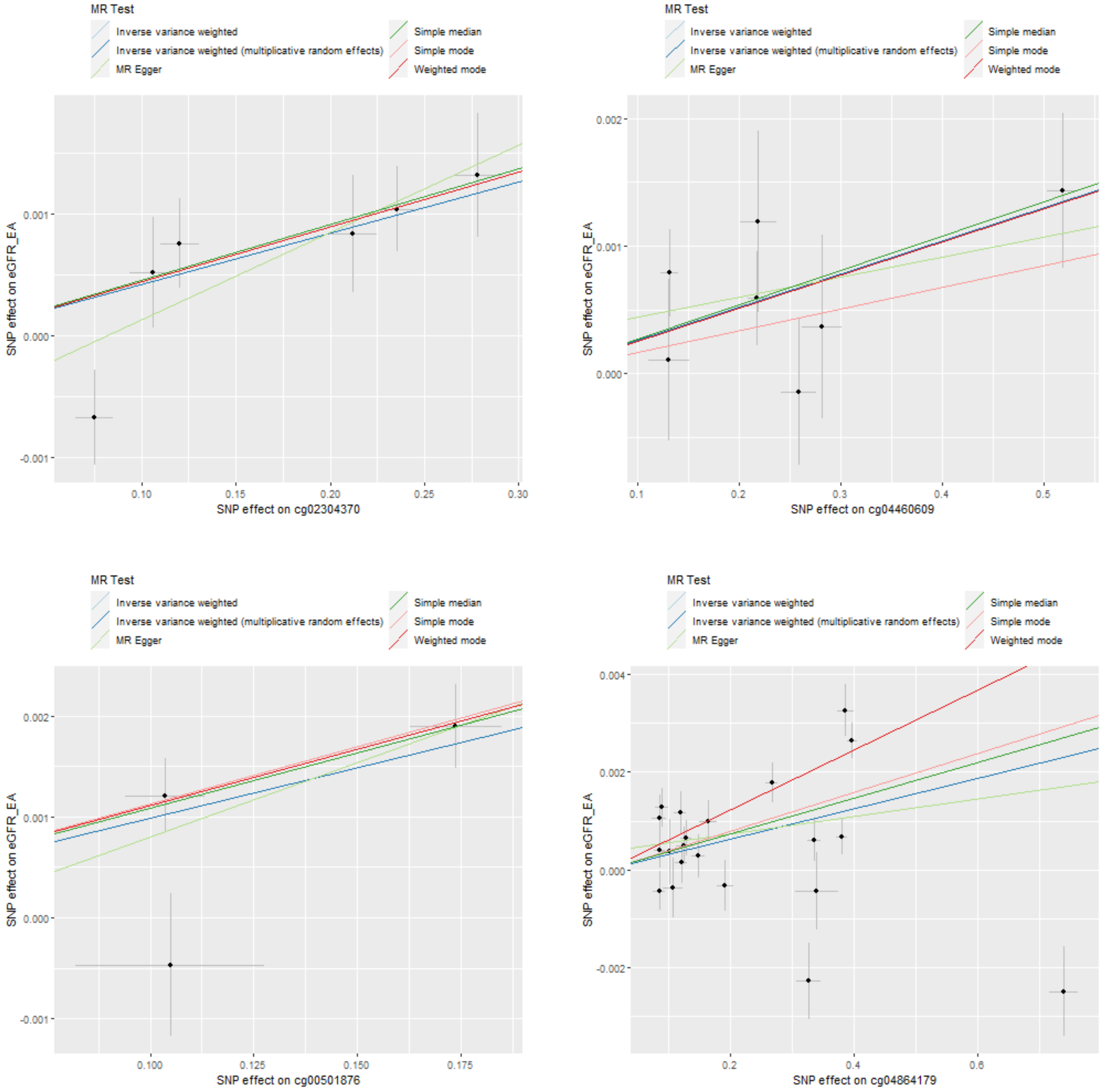

B

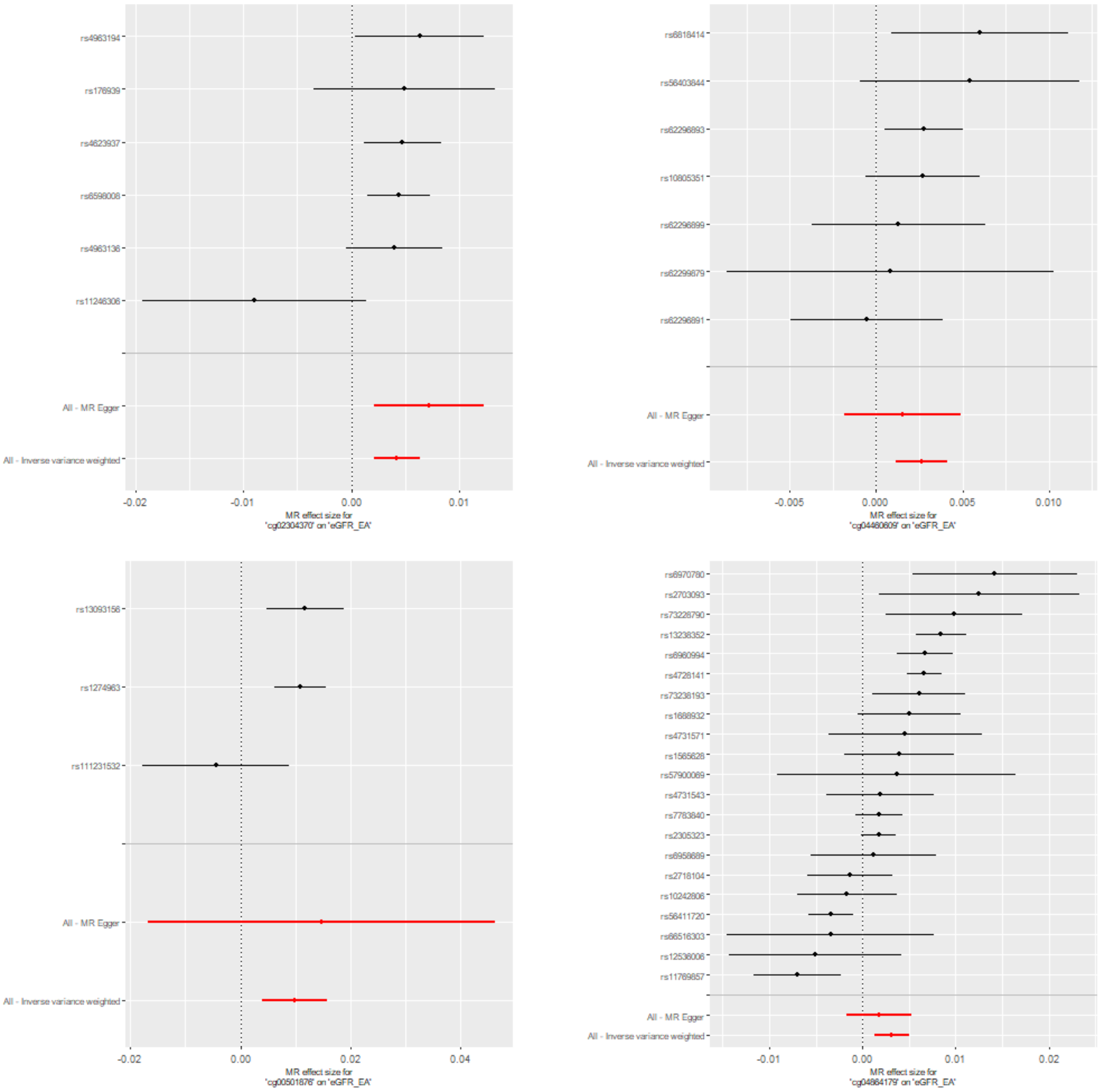

Scatter plots (A) and forest plots (B) of the respective two-sample (n_{eGFR}=567,460; n_{DNAm}=27,750) Mendelian randomization (MR) test results of the four CpGs that suggest a causal effect on estimated glomerular filtration rate (GFR). In panel A, the effects of the x- and y-axis are obtained from the association test of the respective SNP on eGFR and DNA methylation, respectively. In panel B, the effect sizes of the individual SNPs represent the triangulation of their association result on eGFR and DNA methylation. In both panels, the error bars indicate the 95% confidence interval of the corresponding effect size.

Supplementary Figure 8: BACON plots

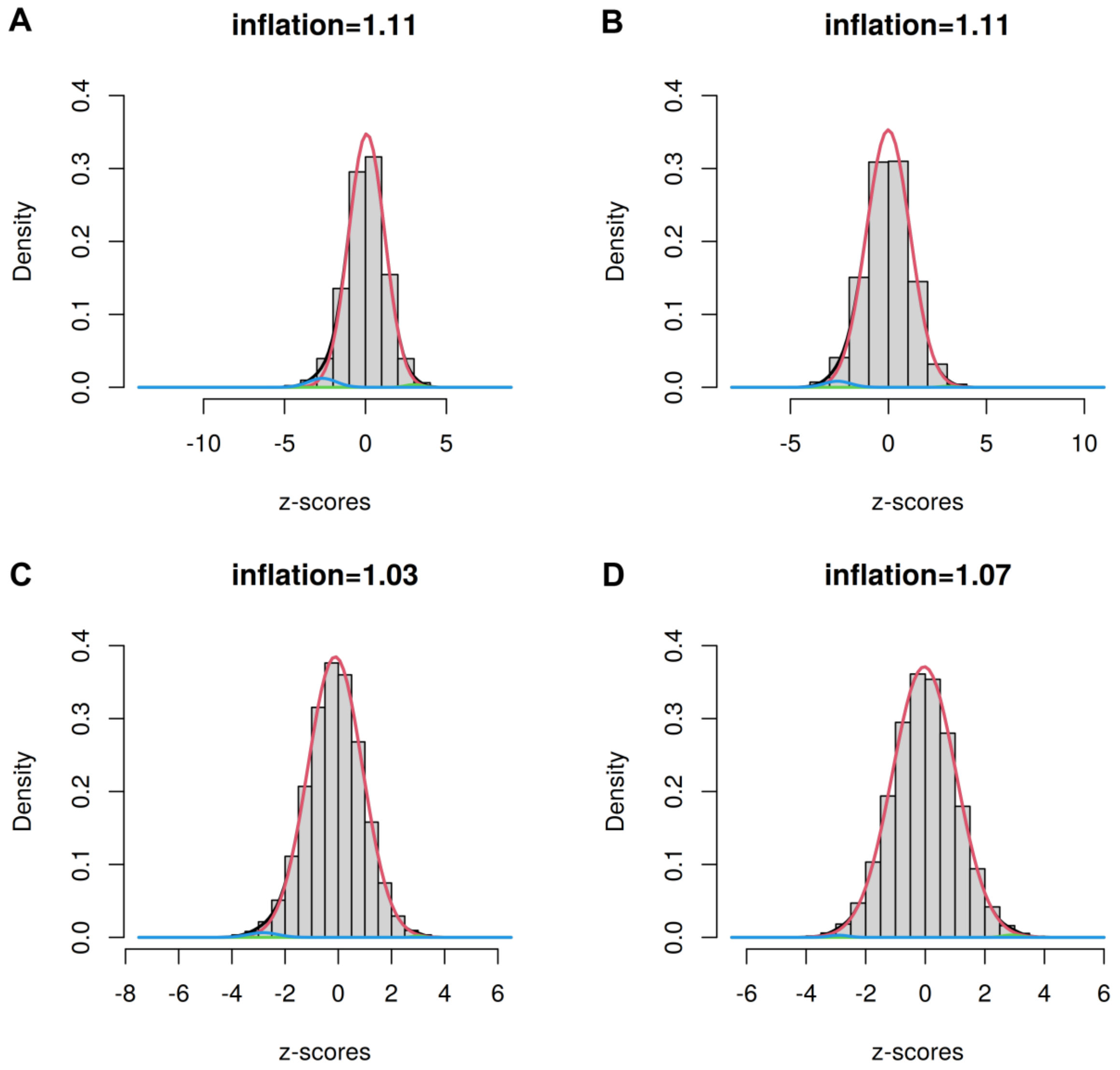

BACON plot from the trans-ethnic meta-analysis of epigenome-wide association studies of estimated glomerular filtration rate (A), chronic kidney disease (B), urinary albumin-to-creatinine ratio (C) and microalbuminuria (D). Histogram of standardized effect estimates. Black line represents to overall fit, red the fit of the null distribution, and blue and green the alternatives.

Supplementary Figure 9: Schoenfeld residuals of the time-to-event analyses in CKD patients

A

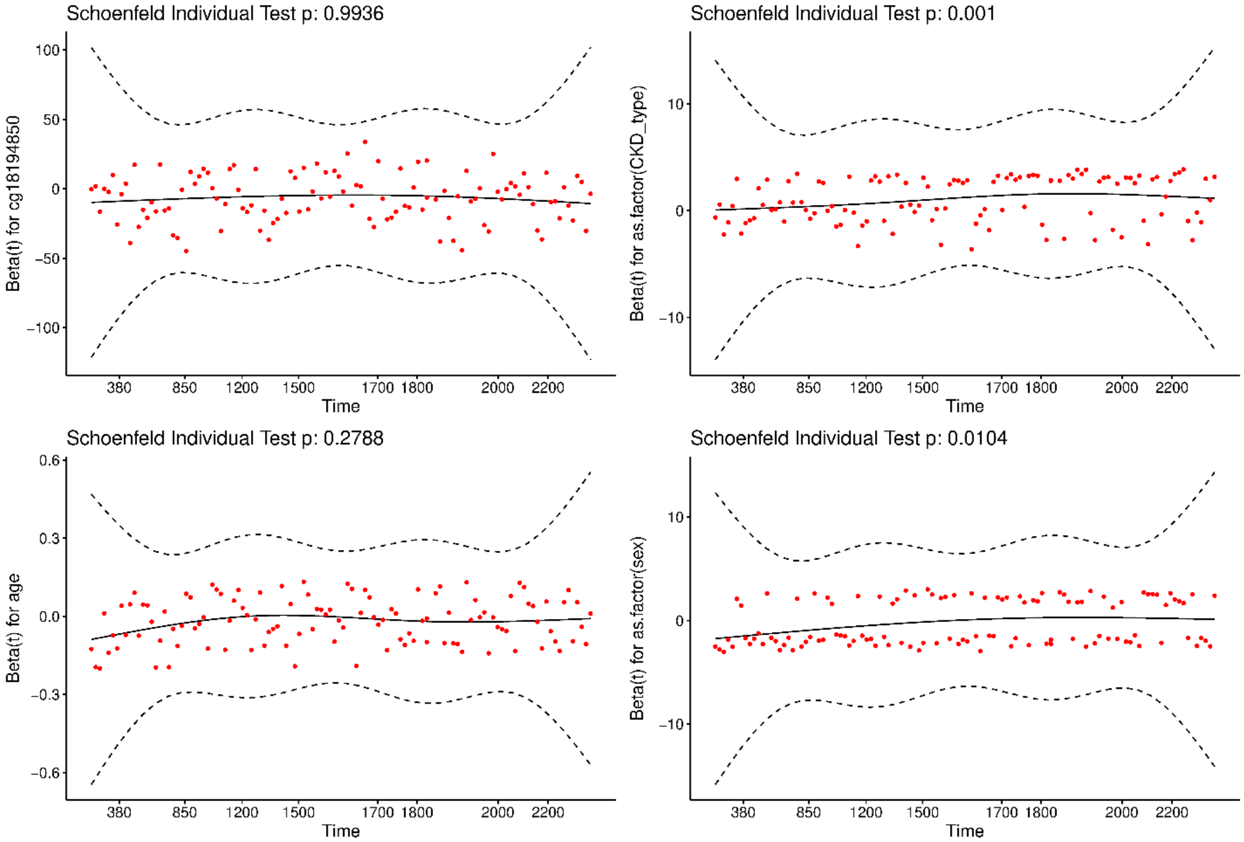

B

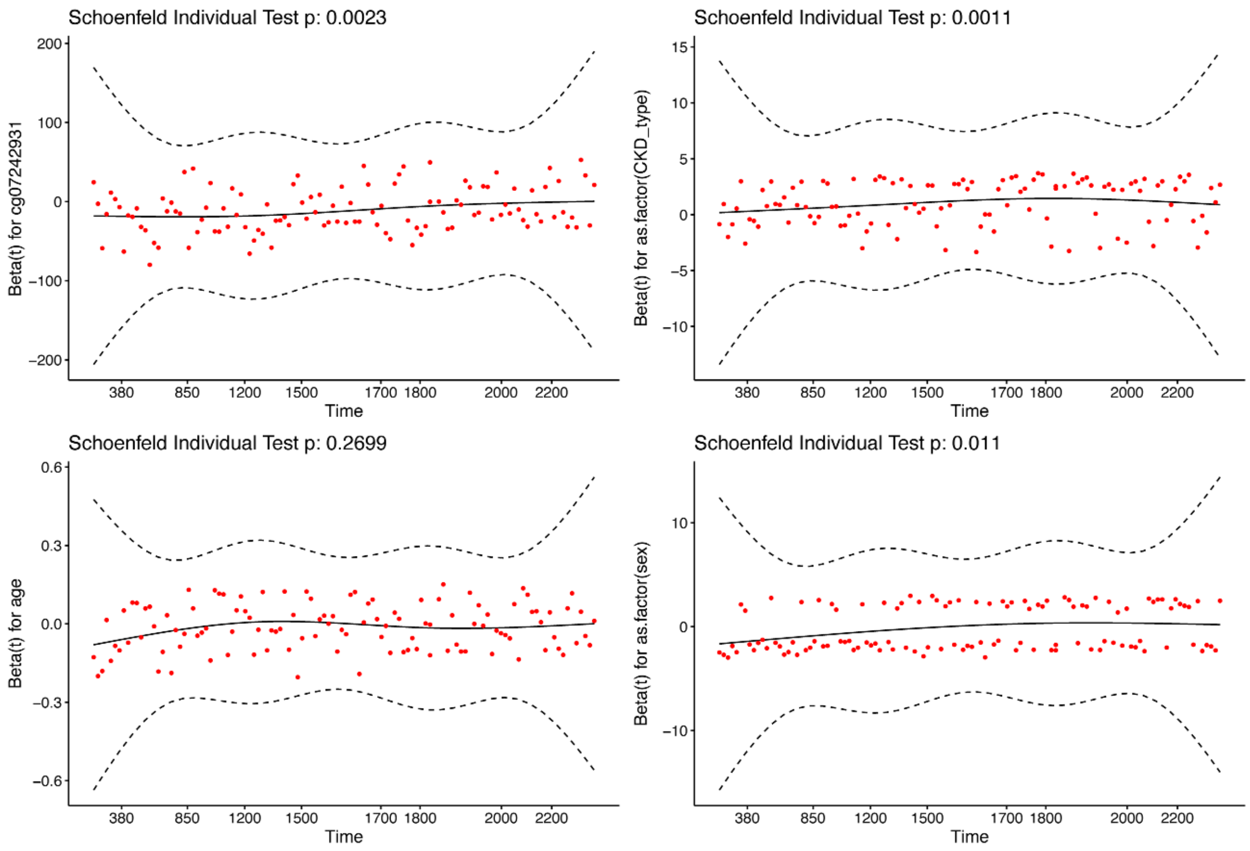

The Schoenfeld residuals of the Cox regression on cg18194850 (A) and cg07242931 (B) in the GCKD cohort.
