# Supplementary material for: Meta-analyses identify DNA methylation associated with kidney function and damage
Source: Nat Commun. 2021 Dec 9;12:7174. doi: 10.1038/s41467-021-27234-3 (PMC8660832; doi:10.1038/s41467-021-27234-3)
Supplement: Supplementary file 3 — Description of Additional Supplementary Files [file 41467_2021_27234_MOESM3_ESM.pdf]

Description of Supplementary Files

File Name: Supplementary Data 1
Description: Population characteristics of participating eGFR/CKD studies

File Name: Supplementary Data 2
Description: Population characteristics of participating UACR/microalbuminuria studies

File Name: Supplementary Data 3
Description: Cohort-specific DNA methylation methods

File Name: Supplementary Data 4
Description: Replicated CpGs from meta-analysis of epigenome-wide association studies of eGFR

File Name: Supplementary Data 5
Description: Replicated CpGs from meta-analysis of epigenome-wide association studies of UACR

File Name: Supplementary Data 6
Description: Suggestive CpGs from meta-analysis of epigenome-wide association studies of eGFR

File Name: Supplementary Data 7
Description: Suggestive CpGs from meta-analysis of epigenome-wide association studies of UACR

File Name: Supplementary Data 8
Description: Competing events analysis for time to kidney failure or acute kidney injury

File Name: Supplementary Data 9
Description: Association between the eGFR/UACR-associated CpGs and gene expression

File Name: Supplementary Data 10
Description: Association between eGFR/UACR-associated CpGs and eGFR/fibrosis in kidney tubule tissue

File Name: Supplementary Data 11
Description: Results of the forward Mendelian randomization analysis

File Name: Supplementary Data 12
Description: Results of the reverse Mendelian randomization analysis

File Name: Supplementary Data 13
Description: Enrichment analysis of eGFR-associated CpGs in transcription factor binding sites

File Name: Supplementary Data 14
Description: Enrichment analysis of UACR-associated CpGs in transcription factor binding sites

File Name: Supplementary Data 15
Description: Enrichment analysis of eGFR-associated CpGs for histone marks

File Name: Supplementary Data 16
Description: Enrichment analysis of UACR-associated CpGs for histone marks

File Name: Supplementary Data 17
Description: Gene set enrichment analysis of eGFR-associated CpGs in GO / KEGG / Reactome

File Name: Supplementary Data 18
Description: Gene set enrichment analysis of UACR-associated CpGs in GO / KEGG / Reactome

File Name: Supplementary Data 19
Description: EWAS catalog look up for eGFR/UACR-associated CpGs
